# Natural variation in tolerance to sub-zero temperatures among populations of *Arabidopsis lyrata *ssp. *petraea*

**DOI:** 10.1186/s12870-018-1513-0

**Published:** 2018-11-12

**Authors:** Matthew P. Davey, Ben G. Palmer, Emily Armitage, Philippine Vergeer, William E. Kunin, F. Ian Woodward, W. Paul Quick

**Affiliations:** 1 Current address: Department of Plant Sciences, Downing Street, Cambridge, CB2 3EA UK; 2 Animal and Plant Sciences, Western Bank, University of Sheffield, Sheffield, UK; 3 Plant Ecology and Nature Conservation Group, Wageningen University, Wageningen, The Netherlands; 4 Institute of Integrative and Comparative Biology, University of Leeds, Leeds, UK

**Keywords:** Acclimation, *Arabidopsis lyrata* ssp.* petraea*, Chlorophyll fluorescence, Marginal populations, Survival

## Abstract

**Background:**

Temperature is one of the most important abiotic factors limiting plant growth and productivity. Many plants exhibit cold acclimation to prepare for the likelihood of freezing as temperatures decrease towards 0 °C. The physiological mechanisms associated with enabling increased tolerance to sub-zero temperatures vary between species and genotypes. Geographically and climatically diverse populations of *Arabidopsis lyrata *ssp. *petraea* were examined for their ability to survive, maintain functional photosynthetic parameters and cellular electrolyte leakage integrity after being exposed to sub-zero temperatures. The duration of cold acclimation prior to sub-zero temperatures was also manipulated (2 and 14 days).

**Results:**

We found that there was significant natural variation in tolerances to sub-zero temperatures among populations of *A. petraea*. The origin of the population affected the acclimation response and survival after exposure to sub-zero temperatures. Cold acclimation of plants prior to sub-zero temperatures affected the maximum quantum efficiency of photosystem II (PSII) (*F*_*v*_/*F*_*m*_) in that plants that were cold acclimated for longer periods had higher values of *F*_*v*_/*F*_*m*_ as a result of sub-zero temperatures. The inner immature leaves were better able to recover *F*_*v*_/*F*_*m*_ from sub-zero temperatures than mature outer leaves. The Irish population (Leitrim) acclimated faster, in terms of survival and electrolyte leakage than the Norwegian population (Helin).

**Conclusion:**

The ability to survive, recover photosynthetic processes and cellular electrolyte leakage after exposure to sub-zero temperatures is highly dependent on the duration of cold acclimation.

## Background

Variation in freezing tolerance and the effectiveness of cold acclimation of plants before exposure to sub-zero temperatures varies within and between species. Furthermore, this variation is pronounced along latitudinal and altitudinal gradients, such that variation in response to low and sub-zero temperatures should be expected between species and genotypes which are geographically distant [1, 2]. This response could also be affected by the length of time plants are cold acclimated prior to sub-zero temperatures in nature [3, 4]. The ability of plants to acclimate to cold temperatures is related to a variety of metabolic and molecular processes [5, 6] such as those involved in maintaining or improving the integrity of the photosynthetic apparatus and cell membranes [7–9]. The photosynthetic apparatus can be severely affected by temperature [10, 11]. For example, the rate at which electrons can be transported along the electron transport chain is decreased at low temperatures [12, 13]. A decrease in photosystem II (PSII) activity can also occur at low temperatures [14, 15] and can be measured using chlorophyll fluorescence techniques that determine the maximum quantum efficiency of PSII (*F*_*v*_/*F*_*m*_) [16–18]. The recovery of cold induced decreases in *F*_*v*_/*F*_*m*_ can vary according to genotypes within a plant species [19]. More recently, changes in chlorophyll fluorescence have been used to accurately quantify freezing damage in two ecotypes of the model species *Arabidopsis thaliana* [20]. Ehlert and Hincha [20] also used chlorophyll fluorescence imaging, linked to electrolyte leakage, to observe the spatial changes in *F*_*v*_/*F*_*m*_ of cold acclimated and non-acclimated, excised leaves of *A. thaliana*. There is an advantage of imaging *F*_*v*_/*F*_*m*_ in that the spatial changes in response to a potentially lethal treatment can be observed [21].

**Fig. 1:**
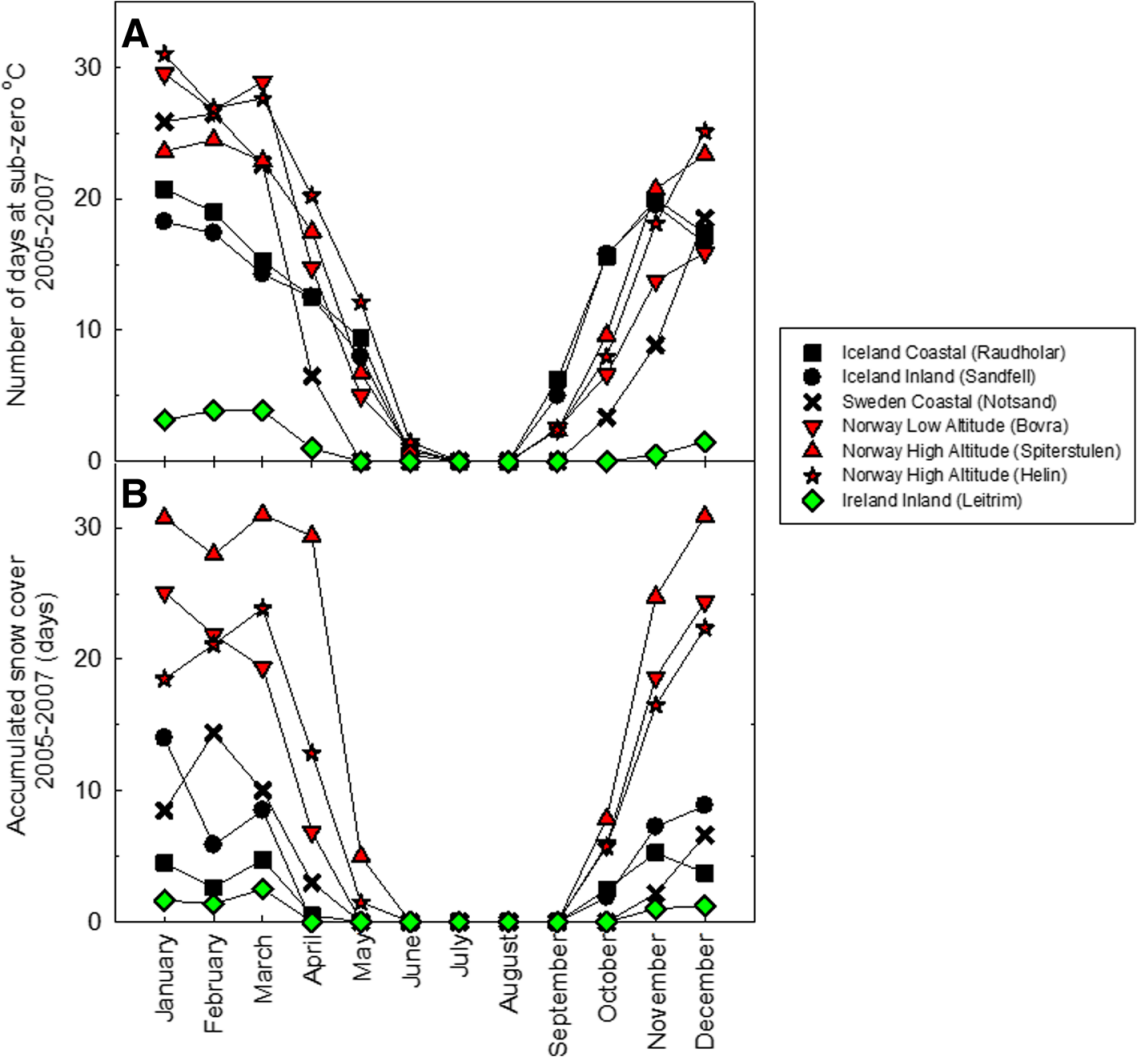
**a**: The number of days where the minimum temperature dropped below zero degrees Celsius for each site. **b**: The number of days with snow cover defined as the number of days with a variation in daily (24 h) temperature of less than 2 degrees Celsius and with temperatures constantly below 1 degree Celsius. Temperature recordings were taken every 120 min over a period of two years (August 2005 – August 2007)

**Fig. 2:**
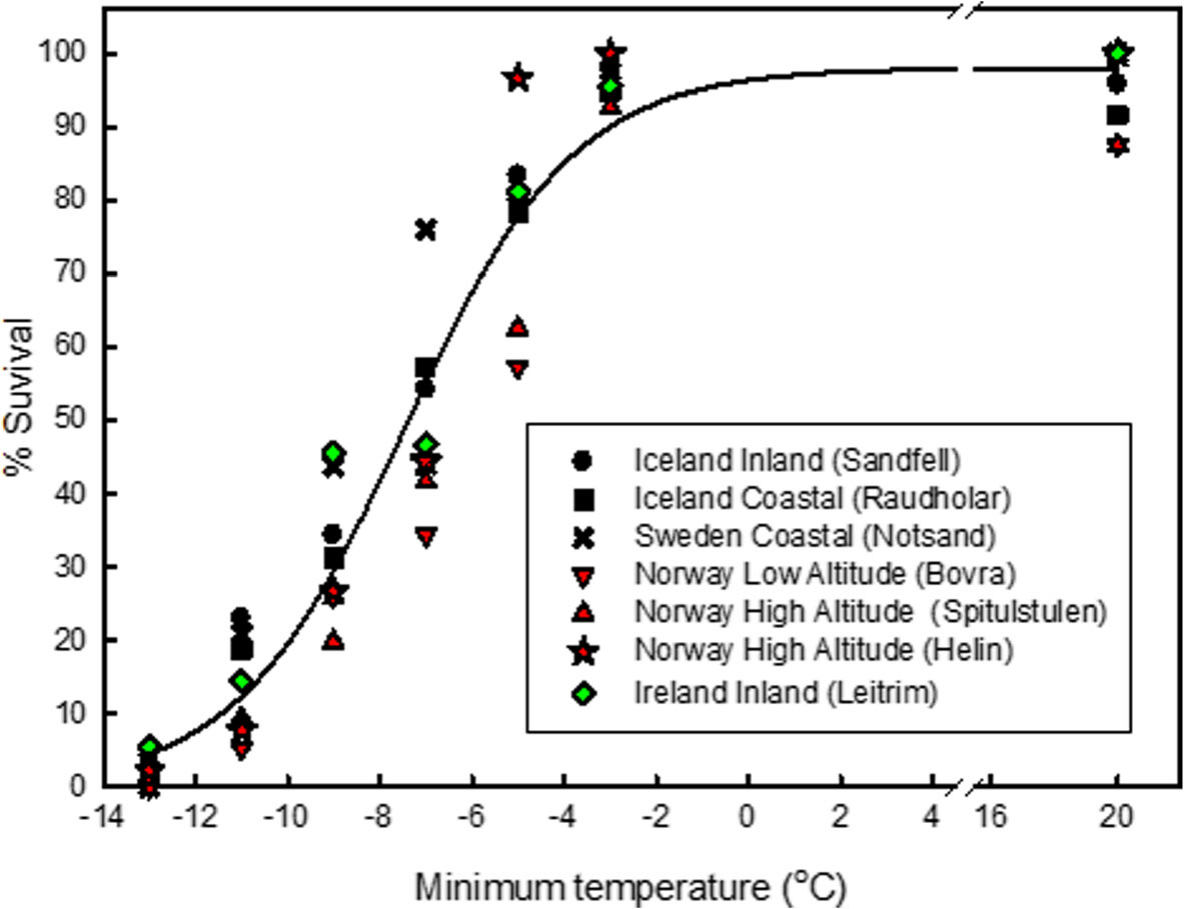
Percent survival of *Arabidopsis lyrata *ssp. *petraea* from separate populations, 14 days after exposure to sub-zero temperatures. Plants were exposed to 2 °C for 2 days prior to exposure to sub-zero temperatures. *n* = 90 to 96 apart from 20 °C where *n* = 24. Curve represents mean survival of all populations. LT_50_ values are presented in Table 1

Alongside values of *F*_*v*_/*F*_*m*_, studies on the natural variation in freezing tolerance for *A. thaliana* have demonstrated a correlation between survivorship values (lethal temperature at 50% survival, LT_50_) and habitat minimum temperature [22], of which the genetic and molecular basis of this variation has been well characterised [23, 24]. However, unlike the annual species *A. thaliana*, cold acclimation and tolerance to sub-zero temperatures is especially critical to survival in perennial Arctic-Alpine plants as they are exposed to a wider range of temperatures over the year. Phenotypic, genetic and metabolic differences have been reported between geographically isolated populations of the Arctic-Alpine perennial *Arabidopsis lyrata. *ssp. *petraea* (hereafter *A. petraea*) [25–29]. Therefore, we hypothesised that there would be inherent differences in other phenotypic characteristics that are associated with changes in cold acclimation duration and exposure to sub-zero temperatures, namely the maximum efficiency of photosystem II (*F*_*v*_/*F*_*m*_) and the integrity of cellular membranes (electrolyte leakage) among the populations of this species. To this end, geographically and climatically diverse populations of *A. petraea* were examined for their ability to survive, maintain functional photosynthetic parameters and avoid cellular electrolyte leakage after being exposed to sub-zero temperatures following a period of cold temperature (acclimation). Plants from the Norwegian high altitude population (Helin) and the Irish low altitude population (Leitrim) were selected for further study due to the large differences experienced by these two populations in mean annual temperatures (Table 1); the number of days recorded at sub-zero temperatures and snow cover (Fig. 1) and the percent survival of plants at sub-zero temperatures (Fig. 2). It was also hypothesised that plants from the Norwegian population would exhibit greater tolerance to sub-zero temperatures than plants from the Irish population. The treatment temperature of − 9 °C was chosen for single sub-zero temperature exposures for % survival, *F*_*v*_/*F*_*m*_ and electrolyte leakage measurements as this was the temperature at which there was the greatest difference between the percent survival of Leitrim and Helin plants (Fig. 2).

**Table 1:** Location and altitude for each population of *Arabidopsis lyrata *ssp.* petraea* in this study

Country	Population	Latitude (decimal degrees)	Longitude (decimal degrees)	Altitude (masl)	Average yearly site temperature (°C)	Site description	LT_50_ (°C)
Iceland	Raudholar	64.093	21.749	90	5.0	Coastal	−7.8
Iceland	Sandfell	64.073	21.683	119	4.7	Inland, low altitude	−7.7
Sweden	Notsand	62.609	18.062	3	7.5	Coastal, low altitude	−8.6
Norway	Bovra	61.770	8.419	493	4.6	Low altitude	−6.2
Norway	Spiterstulen	61.658	8.427	953	3.6	High altitude	−6.5
Norway	Helin	61.046	8.666	1141	4.5	High altitude	−7.4
Ireland	Leitrim	54.383	8.377	355	8.8	Inland, low altitude	−7.7

*Masl* metres above sea level. Lethal temperature for 50% survival after exposure to sub-zero temperatures after two days cold acclimation at 2 °C (LT_50_)

## Results

### Climate data

Across all the sites (Table 1), there was large variation in the number of days where the minimum temperature dropped below zero degrees Celsius and in the number of days with snow cover (Fig. 1). The Leitrim site (Ireland) had the fewest days at sub-zero temperatures and the least number of days with snow cover. The sites in Norway had the greatest number of days at sub-zero temperatures and with snow cover, followed by Sweden and then Icelandic sites.

### Short term (2 days) cold acclimation

The percent survival of plants exposed to sub-zero temperatures was dependent on the plants’ geographic origin (Fig. 2). There was a difference of 2.4 °C between the lowest and highest lethal temperature for 50% death (LT_50_) for the populations of *A. petraea* (Sweden, Notsand − 8.6 °C and Norway low altitude (Bovra) -6.2 °C, respectively) (Table 1). The temperature at which there was the largest differential in survival was − 7 °C where 76% of the Swedish coastal (Notsand) population survived while only 34% of the Norwegian low altitude (Bovra) population survived. The largest difference between survival for the targeted populations occurred at − 9 °C where 46% of the Irish (Leitrim) population survived while only 27% of the Norwegian high altitude (Helin) population survived.

### Maximum efficiency of photosystem II (*F*_*v*_*/F*_*m*_)

**Fig. 3:**
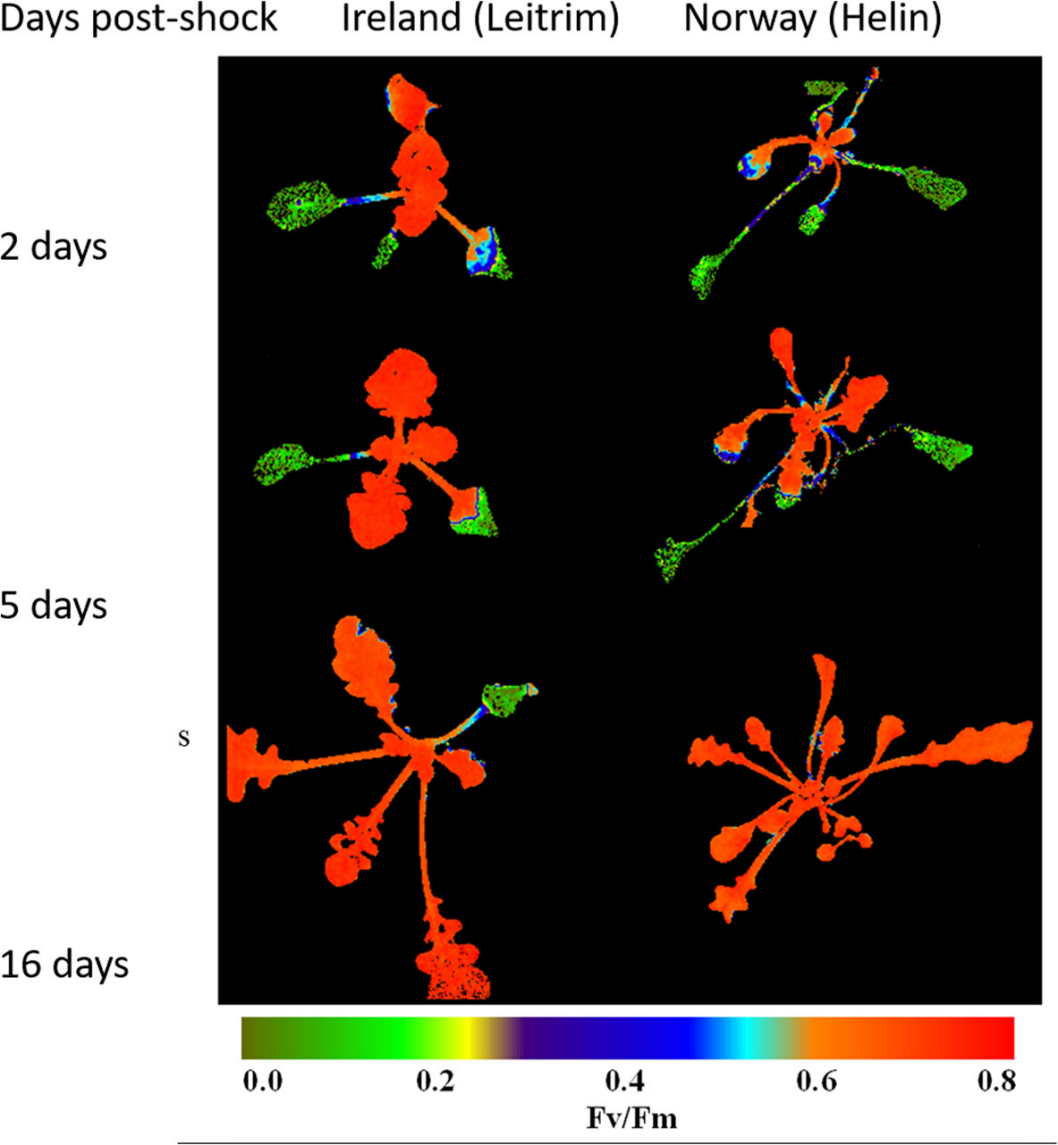
Spatial changes in the maximum efficiency of photosystem II (*F*_*v*_/*F*_*m*_) of *Arabidopsis lyrata *ssp. *petraea* seedlings. Images represent the recovery of *F*_*v*_/*F*_*m*_ in a single representative plant 2, 5 and 16 days after − 9 °C treatment (false colour imaging) from the Irish (Leitrim) and Norwegian (Helin) populations. Prior to sub-zero treatment, plants were exposed to 2 °C for 2 days

In Norwegian (Helin) and Irish (Leitrim) populations, there were spatial differences in the recovery of the maximum efficiency of photosystem II (*F*_*v*_/*F*_*m*_) (Fig. 3). The ability of the inner leaves to recover *F*_*v*_/*F*_*m*_ and survive sub-zero temperatures is shown by *F*_*v*_/*F*_*m*_ images taken 2, 5 and 16 days post sub-zero treatment of the same plant for each population (Fig. 3). The surviving inner leaves have grown whereas the older damaged leaves died after 16 days. There was a substantial between-population difference with the Irish population maintaining *F*_*v*_/*F*_*m*_ in more leaves (mature and immature) compared to the Norwegian population.

**Fig. 4:**
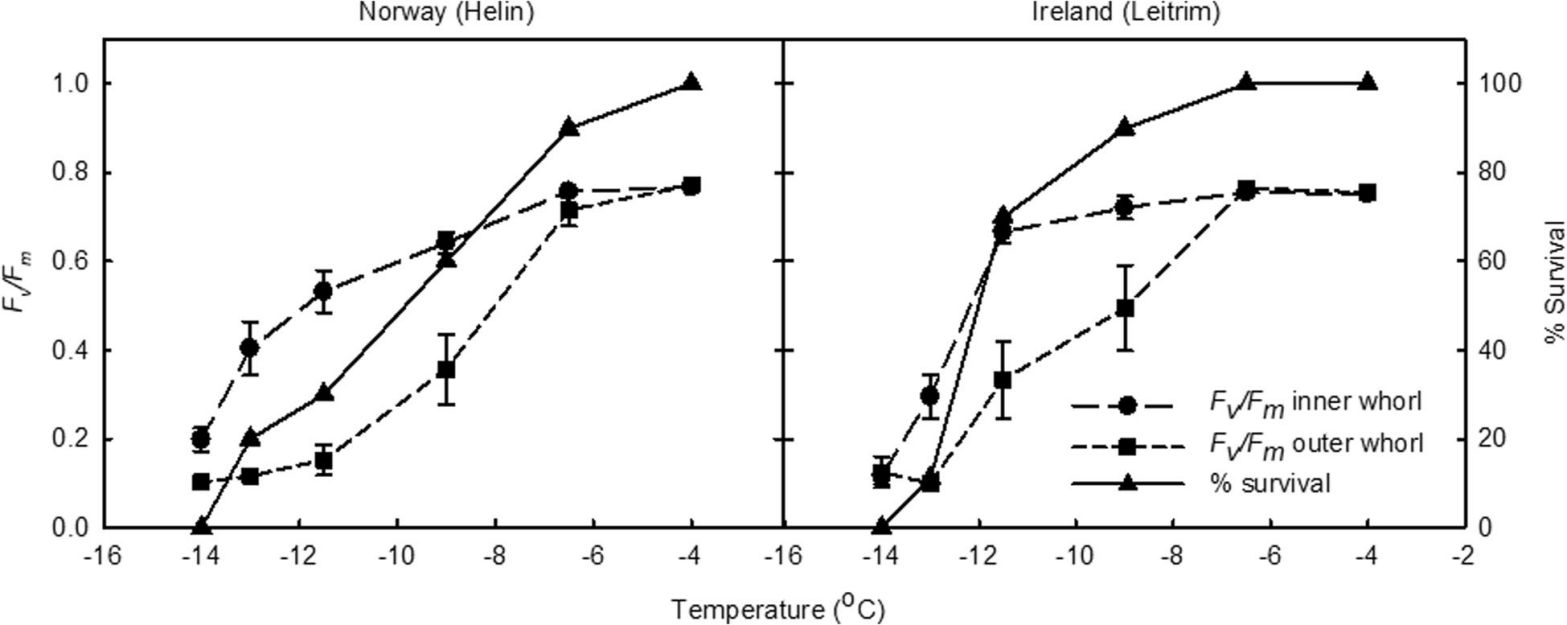
Maximum efficiency of photosystem II (*F*_*v*_*/F*_*m*_) of *Arabidopsis lyrata *ssp. *petraea* seedlings (10 leaves) 2 days after sub-zero treatments. The *F*_*v*_*/F*_*m*_ of inner leaves is defined as the central immature, inner whorl leaves and the outer leaves defined as the larger outer whorl mature leaves. Prior to treatment, plants were exposed to 2 °C for two days. The percent survival of the plants was assessed 16 days after exposure to sub-zero temperatures. *n* = 10 ± SE

To qualify whether the inner whorl recovery was responsible for final plant survival after the sub-zero treatments, the average *F*_*v*_/*F*_*m*_ values of the immature inner whorl and mature outer whorl leaves of each plant was obtained by selecting such regions in each image. This confirmed that there was a difference in *F*_*v*_/*F*_*m*_ values between the inner immature leaves and the outer mature leaves (Fig. 4). The final percent survival of the two populations differed between the two populations, with plants from the Irish population able to survive lower temperatures (Fig. 4). The survival of the Irish plants was correlated to the *F*_*v*_/*F*_*m*_ values of the inner leaves (*F*_*v*_/*F*_*m*_ > 0.5) as the sharp decrease in *F*_*v*_/*F*_*m*_ at − 13 °C was related to a severe decrease in the number of surviving plants. This pattern was not replicated in the outer mature leaves. Similarly in the Norwegian plants, the survival of the plants was related to maintaining *F*_*v*_/*F*_*m*_ values of the inner leaves as the *F*_*v*_/*F*_*m*_ of the outer leaves values were less than 0.2 from − 11.5 °C onwards.

### Short and long term (2 and 14 days) cold acclimation

**Fig. 5:**
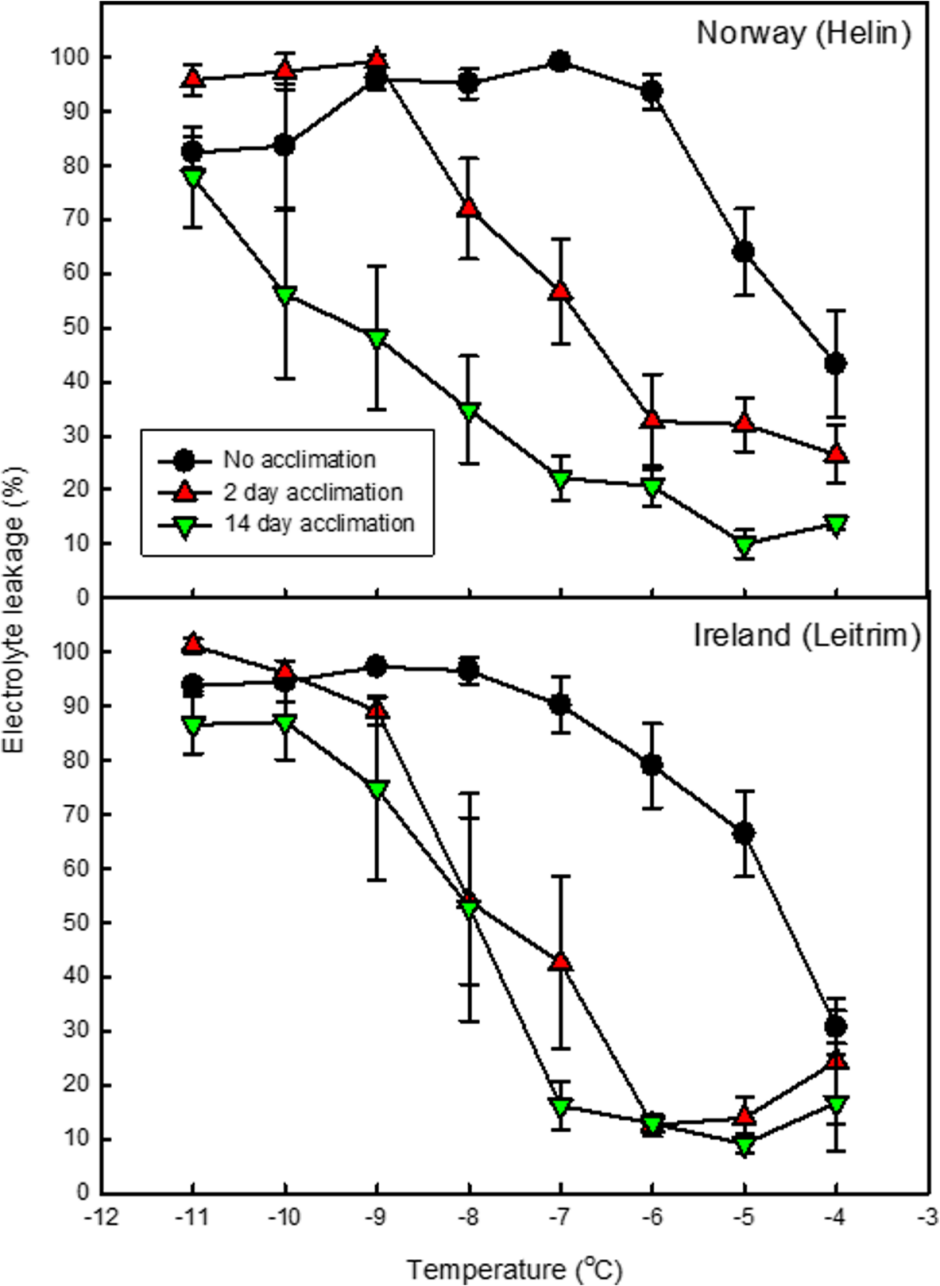
Cellular electrolyte leakage (%) of excised mature leaves from two individual populations of *Arabidopsis lyrata *ssp. *petraea* after exposure to a range of sub-zero temperatures. Sub-zero temperatures applied after no acclimation or 2 or 14 days acclimation at 2 °C. *n* = 4 ± SE. TEL_50_ values are presented in Table 2

An increase in cold acclimation duration, from 0 to 2 and then 14 days, resulted in an increase in the number of plants surviving − 9 °C temperatures (Table 2). After 2 days of cold acclimation, more Irish plants survived than Norwegian plants. However, after 14 days acclimation, both populations had a similar number of plants surviving the sub-zero temperatures. The increase in survivorship with increased acclimation duration was reflected in the changes in *F*_*v*_/*F*_*m*_ and electrolyte leakage results (Table 2; Fig. 5) where after 2 days cold acclimation, the *F*_*v*_/*F*_*m*_ was significantly greater in plants from Ireland (Leitrim), compared to those from Norway (Helin). There was no significant difference in *F*_*v*_/*F*_*m*_ between populations in plants exposed to 14 days cold acclimation prior to a − 9 °C treatment (Table 2).

**Table 2:** Percent survival, maximum efficiency of photosystem II (*F*_*v*_/*F*_*m*_) and electrolyte leakage at − 9 °C and temperature for 50% electrolyte leakage (TEL_50_) of two individual populations of *Arabidopsis lyrata* ssp.* petraea* after exposure to sub-zero temperatures for one night

	Cold acclimation duration (days)	Helin (Norway)	Leitrim (Ireland)
% survival after exposure to − 9 °C	0	0	8
	2	27	46
	14	81	76
*F*_*v*_/*F*_*m*_ after exposure to − 9 °C	2	0.35 (± 0.06)	0.57 (± 0.04) **
	14	0.43 (± 0.02)	0.48 (± 0.03) ns
Electrolyte leakage (%) at −9 °C	0	96.0 (± 2.0)	97.3 (± 0.2) ns
	2	99.3 (± 1.0)	89.2 (± 2.6) *
	14	48.2 (± 13.0)	74.8 (± 17.0) ns
TEL_50_ (°C)	0	−4.2	−4.5
	2	−6.7	−7.6
	14	−9.2	−7.8

Sub-zero temperatures were applied at a rate of − 2 °C per hour after 0, 2 or 14 days cold acclimation at 2 °C. Statistically significant differences between populations are indicated by * = *P* ≤ 0.05; ** = *P* ≤ 0.01; ns = not significant

Similarly, there were contrasting cellular electrolyte leakages of these two populations when exposed to sub-zero temperatures and different cold acclimation durations (Table 2). The cellular electrolyte leakage was significantly lower in the Irish plants exposed to − 9 °C after a 2 day cold acclimation period, compared to Norway plants. However, in plants exposed to 14 days cold acclimation, the electrolyte leakage was substantially lower in Norway plants compared to Ireland, but this difference was not statistically significant. Increased acclimation duration in the Irish population resulted in the temperature at which 50% electrolyte leakage occurs (TEL_50_) decreasing from − 4.5 °C in the control samples with no prior cold acclimation to − 7.6 °C in 2 day acclimated samples to − 7.8 °C in 14 day acclimated samples. This reduction in leakage was even more pronounced in the Norwegian population where the TEL_50_ of electrolyte leakage was reduced from − 4.2 °C in the no acclimation samples to − 6.7 °C in 2 day acclimated samples to − 9.2 °C in 14 day acclimated samples.

## Discussion

This study has shown that there is significant intraspecific variation in freezing tolerance, chlorophyll fluorescence and cellular electrolyte leakage in response to sub-zero temperatures. There were significant differences in the ability of plants from different geographic origins to survive sub-zero temperatures. Despite the fact that average annual temperatures for the Swedish populations were higher than the Norwegian populations (Table 1), the Swedish population (Notsand) had the greater tolerance to sub-zero temperatures (TEL_50_ at − 8.6 °C), whereas a Norwegian population (Bovra) had the lowest tolerance (TEL_50_–6.2 °C) (Fig. 1). One explanation could be that although plants from Norwegian and Swedish populations would experience a similar number of days below zero degrees C when grown in the field, the number of days of snow cover measured in the Norwegian populations was greater than the Swedish sites (Fig. 1). This would allow the Swedish population to have a longer cold acclimation period, favouring plants with a higher tolerance to freezing. However, other factors may be the cause of the difference, such as coastal humidity or the absence of snow cover in a cold period may cause soils to freeze to greater depths and expose plants to greater physical and physiological stress [30]. This snow cover would thus provide greater insulation for the Norwegian plants whilst the Swedish plants would be exposed to colder, sub-zero, temperatures. The majority of plants did not survive temperatures below − 10 °C, however, those that did were mostly from populations with low snow cover sites (Iceland, Ireland and Sweden). Such variation in the number of plants able to survive such sub-zero temperatures will be critical in establishing and maintaining populations, especially in marginal populations where the inherent ability to survive sub-zero temperatures outside the population norm would be critical in expanding the population.

The detailed studies on the Leitrim (Irish) and Helin (Norwegian) populations revealed population-specific responses to cold acclimation duration and sub-zero temperatures. More plants from the Irish population were able to survive sub-zero temperatures than the Norwegian population, especially when acclimated for 2 days. That plants from warmer locations would be more tolerant to sub-zero temperatures than those originating from colder locations was counter to our expectations. One explanation could be that this is due to adaptive phenotypes obtained from a historical climate which are still conserved in the population [31]. However, this effect was somewhat reversed, albeit not significantly so, when plants were acclimated for 14 days. Therefore, cold acclimation and survival duration is critical for young seedlings prior to a freezing event, such as that experienced on a clear frosty night [32]. Alternatively, as plants in the Irish sites would not have experienced sub-zero conditions during the summer and most of the autumn months, they may have adapted to acclimate faster for an approaching winter. This is in contrast to the Norwegian plants where summer and autumn nightime temperatures regularly fall to below zero degree C (Fig. 1) and do not clearly indicate the approach of winter.

Chlorophyll fluorescence measures the state of photosystem II (PSII) such that if it is damaged, *F*_*v*_/*F*_*m*_ may be reduced. This indicates that damage from sub-zero temperatures is lessened with an increase in cold acclimation and plants become more low temperature and sub-zero tolerant. It was evident from the *F*_*v*_/*F*_*m*_ imaging that the Irish population had a greater area of maintained PSII in the inner immature leaves and the outer mature leaves than the Norwegian population (Fig. 3), and that this was related to recovery and survival of the plants (Fig. 4). This is similar to results in Demmig-Adams [16], Haldimann et al. [33, 34] where leaves of cold acclimated herbaceous plants exhibit a substantially decreased susceptibility to photoinhibition, possibly caused by a greater capacity to dissipate energy and increase carotenoid concentrations such as zeaxanthin. That inner younger leaves are more likely to survive cold temperature shocks suggests that developmental processes are central to the acclimation process and likely to involve morphological and metabolic changes that are not possible in mature leaves [35]. Improved acclimation at 14 days rather than 2 days may therefore just reflect the number of leaves that were developing during the acclimation period. Another explanation is that the *F*_*v*_/*F*_*m*_ was an indirect measure for damage (and subsequent recovery) of the cell membranes (measured as electrolyte leakage).

The measurements of electrolyte leakage were in line with other reports on cold acclimation in *Arabidopsis thaliana* genotypes [9, 36]. Observations of electrolyte leakage indicated two different strategies for cold acclimation, a fast and a slow acclimator, where plants from the Helin population were slower to alter their cell membrane properties than those from the Leitrim population (Fig. 5; Table 2). However, overall the plants from Helin had less cellular electrolyte leakage after 14 days of cold acclimation (48% leakage; TEL_50_ of − 9.2 °C), than plants from the Leitrim population (74.8%; TEL_50_ of − 7.8 °C). Although the electrolyte leakage measurements were not directly correlated with the final percent survival of the populations at various temperatures, these data show that the Helin population is able to withstand colder temperatures better than the Leitrim population, so long as the plants have had a long cold acclimation period.

## Conclusion

These findings show that there is significant natural variation in tolerances to sub-zero temperatures among populations of *A. petraea*, which may signify plant adaptation to local climates. It was hypothesised that acclimation of plants prior to sub-zero temperatures would have an effect on chlorophyll fluorescence, and that the origin of the population would have an effect on acclimation response and survival after exposure to sub-zero temperatures. This was seen to be the case. However, as only *F*_*v*_/*F*_*m*_ values were measured, it is not clear whether this would be due to changes in the operating efficiency of PSII (Φ_PSII_) or as slowly recovering non-photochemical quenching [37]. The ability of *A. petraea* to survive, maintain functional photosynthetic parameters and cellular electrolyte leakage integrity after being exposed to sub-zero temperatures is likely to be inherently different between the populations. Large differences in phenotype have been reported for *A. petraea* across its Northwest European range [29], as well as genetic differences [25, 26, 38] and metabolite differences between some populations [27, 28]. Our data build on these studies and strongly indicate that differences in cold acclimation, survival and photosynthetic activity recovery may also be inherent among populations of this species.

## Materials and methods

### Climate data

To measure temperature in the field populations, four temperature monitoring probes (Thermocron iButtons Model DS1922L; Maxim Dallas Inc) were placed out in all populations, buried 1 cm below the soil surface to avoid direct radiation exposure. The location of two of these temperature monitoring probes was chosen subjectively to be the warmest and coldest microsites of the site (based largely on slope and aspect). The locations of the other two probes were chosen at random. The probes were always located within 10 cm of an *A. petraea* plant. Temperature recordings were taken every 120 min over a period of 2 years (August 2005 – August 2007), which allowed us to analyse regional microclimate variation in mean and extreme temperatures. The number of days with snow cover was defined as the number of days with a variation in daily (24 h) temperature of less than 1 °C and with temperatures constantly below 1 °C. The number of days where the minimum temperature dropped below 0 °C was also calculated.

### Seed collection and plant growth conditions

Seeds of *Arabidopsis lyrata *ssp. *petraea* were sourced and collected from geographically separated populations within Ireland, Norway, Sweden and Iceland (Table 1). Seed collections were carried out with under permission from all land owners and complied with institutional, national, or international guidelines and legislation. All seeds were half-sibships from the maternal plants. Seeds of each population were sown in Levington M3 compost within individual plug trays. Populations were randomised within each tray and trays were randomly re-positioned every other day. Plants were watered from the base of the pot when required with reverse osmosis (RO) water. No additional nutrients were added to the soil or water. Plants were established in controlled-environment growth cabinets (Conviron Controlled Environments Limited, Canada) set to a 12/12 h day/night cycle; 20/15 °C day/night; 60% humidity, atmospheric CO_2_ concentration ~ 400 ppm CO_2_ and photosynthetically active radiation 250 μmol m^− 2^ s^− 1^. Cold acclimation was achieved by changing the day and night temperature to 2 °C (initially starting from 3 h into the night period, decreasing from 15 °C to 2 °C over 1 h) for either 2 or 14 days prior to sub-zero conditions. Plants that were not cold acclimated remained at 20/15 °C. For sub-zero experiments, seedlings (approximately 2 weeks old) were transferred to a growth room capable of reproducibly ramping and holding sub-zero temperatures prior to the acclimation period (Conviron BDW-40 Controlled Environments Limited, Canada). Sub-zero experiments were carried out during the night cycle as this is when plants mainly experience such temperatures in their natural habitat. After 3 h into the night cycle the temperature was decreased to − 1 °C over 30 min and held at − 1 °C for a further 30 min in order for the growth room temperature to stabilise to the new sub-zero temperatures. The temperature was then decreased at a rate of − 2 °C h^− 1^. After the plants had been exposed to the desired temperature for approximately 10 min, plants were taken out of the growth cabinet and immediately placed into another growth cabinet at 2 °C. After 24 h all plants were subjected to original control conditions (20/15 °C) and percent survival of the plant was measured 14–16 days later to ensure non-ambiguity in whether plants were alive (new tissue forming) or not. Survival curves and LT_50_ values were fitted and calculated using the nonlinear regression - dynamic fitting 3 parameter sigmoidal equations in SigmaPlot v13 (Systat Software Inc., USA).

### Chlorophyll fluorescence

Pre-acclimation and post-sub-zero measurements of chlorophyll fluorescence were taken using a chlorophyll fluorescence imager (Technologica LTD, Colchester). The maximum efficiency of photosystem II (*F*_*v*_/*F*_*m*_) was measured by dark adapting plants for at least 30 min prior to the optimised conditions of a saturating blue light pulse at 3000 μmol m^− 2^ s^− 1^ for 200 ms [39].

### Electrolyte leakage

Larger mature leaves were used for the electrolyte leakage experiment in order to obtain reproducible results and were grown under a 10/14 h day/night cycle at 20 °C. Foliage electrolyte leakage was measured based on the methods by Rohde et al. [40]. Briefly, for each sub-zero temperature point of interest, four fully expanded leaves from four individual plants were placed (one per tube) into a 15 ml plastic centrifuge tube containing 200 μl of distilled water. Tubes were placed into a cooling water bath containing 50% anti-freeze (Ethanediol) set at − 1 °C. After 30 min a few small ice chips were added to each tube to initiate nucleation. After a further 30 min, the samples were cooled at a rate of 2 °C h^− 1^. Tubes were removed at 1 °C intervals and allowed to thaw on ice overnight. Six ml distilled water was added to each tube and shaken overnight at 4 °C in the dark. Once samples had reached room temperature the conductivity (mV) of each sample solution was measured. The samples were then frozen at − 80 °C for 3 h to purge all the cell contents. Once thawed the samples were shaken overnight at 4 °C before final (total) conductivity measurements were made the following day. Percent electrolyte leakage was determined as the ratio of the conductivity measured before freezing at − 80 °C to that after.

### Statistical analyses

XSurvival curves, lethal temperature (LT_50_) and temperature at 50% electrolyte leakage (TEL_50_) values were fitted and calculated using the nonlinear regression - dynamic fitting 3 parameter sigmoidal equations in SigmaPlot v13 (Systat Software Inc., USA). T-test were used to test for significant treatment effects within populations using SPSS v12.0.1 (Chicago, Illinois, USA).

## Abbreviations

*F*_*v*_/*F*_*m*_: Maximum efficiency of photosystem II
LT_50_: Lethal temperature at 50% survival
mV: Conductivity milliVolts
PSII: Photosystem II
RO: Reverse osmosis
TEL_50_: Temperature at 50% electrolyte leakage
Φ_PSII_: Operating efficiency of photosystem II
